# A short peptide potentially promotes the healing of skin wound

**DOI:** 10.1042/BSR20181734

**Published:** 2019-03-22

**Authors:** Yongli Song, Chunyun Wu, Xinghe Zhang, Wenxin Bian, Naixin Liu, Saige Yin, MeiFeng Yang, Mingying Luo, Jing Tang, Xinwang Yang

**Affiliations:** 1 Department of Anatomy and Histology and Embryology, Faculty of Basic Medical Science, Kunming Medical University, Kunming 650500, Yunnan, China; 2 Faculty of Acupuncture and Tuina, Shandong University of Traditional Chinese Medicine, Jinan 250014, Shandong, China; 3 Department of Biochemistry and Molecular Biology, Faculty of Basic Medical Science, Kunming Medical University, Kunming 650500, Yunnan, China

**Keywords:** bioactive peptide, Odorrana andersonii, skin, TGF-β1, wound healing

## Abstract

Skin wound, a common form of skin damage in daily life, remains a serious challenge in clinical treatment. Bioactive peptides with high efficiency have been considered as potential therapeutic candidates for wound healing. In this report, a novel short linear peptide, with mature peptide sequence of ‘GLLSGINAEWPC’ and no obvious similarity with other known bioactive peptides, was identified by genomic method from the skin of odorous frog, *Odorrana andersonii*. Our results suggested that OA-GL12 (OA: abbreviation of species (*O. andersonii*), GL: two initial amino acids, 12: peptide length) obviously accelerated the scratch-healing of keratinocytes and human fibroblasts in a time- and concentration-dependent manner. Meanwhile, OA-GL12 showed significant effect in promoting the wound healing on the full-thickness skin wound model. Inflammatory assay results demonstrated that OA-GL12 induced the secretion of tumor necrosis factor (TNF) and transforming growth factor β1 (TGF-β1) on murine macrophage cell line (RAW264.7), which might explain the powerful accelerating capacity of wound healing. Moreover, results also indicated that epidermal growth factor receptor (EGFR) was involved in the mechanisms underlying the scratch-healing promoting activity of OA-GL12. In addition, OA-GL12 showed obvious free radical scavenging activity. Results supported that OA-GL12 did not exert risk in acute toxicity, hemolytic activity, and direct antibacterial activity. The remarkable effect of OA-GL12 on promoting wound healing verified in this research made it potential to be a novel template for the development of wound healing-promoting agents.

## Introduction

Human skin wounds remain a major and snowballing threat to public health and the economy [1]. Skin is the largest tissue organ covering the human body and the physical barrier between the inner and outer environment. Inevitably, skin wound is often achieved in adverse accidents. Once damaged, the loss of the skin defense against harmful stimulus will contribute to side effects, such as infection, shock, and even death [2–4]. Especially under some conditions (diabetes, infection, et al.), the process of wound healing will be delayed and mostly inflicted chronic wounds [5,6]. Therefore, accelerating the wound healing is vital to the body. Traditional wound healing drugs, including growth factors, cytokines, chemical compounds derived or developed from plants, and other immunomodulatory factors, were particularly difficult to translate these therapies for chronic wound healing into the clinic [7]. Compared with those drugs with high cost, low activity, safety and delivery problems, bioactive peptides with high activity, specificity, and stability have aroused considerable interest in the related field of research [8–10].

Amphibian skins, such as from frogs and toads, are multifunctional organs acting in defense, respiration, and water–salt balance regulation. Because of exposing to sharp rocks, elevated ultraviolet (UV) radiation, and microbes, et al., amphibian skins are susceptible to damage. To defeat these factors, they have evolved a unique and effective polypeptide defense system in their skin secretion, which contains diversity of small bioactive peptides with diverse pharmacological bioactivities, including antimicrobial, antioxidant, and immunomodulatory activities [11]. Particularly, fresh frog skin secretion is efficient in shortening wound healing process in previous researches [12]. Recent studies also reported that bioactive peptides from secretions of odorous frog could promote the wound healing [13,14]. In this study, OA-GL12 (OA: abbreviation of species (*Odorrana andersonii*), GL: two initial amino acids, 12: peptide length), a peptide with excellent wound healing-promoting activity, identified from the skin of *O. andersonii*, a unique branch of odorous frog distributed in southwest China. OA-GL12 showed significant wound healing-promoting ability at actual concentrations both *in vitro* and *in vivo*. Our results suggested that OA-GL12 could be a novel template for the development of wound healing-promoting drug.

## Materials and methods

### Sample collection, animal care, and ethical statement

One adult *O. andersonii* specimens were collected from Yunnan, China. Before the performance of experiments, frog was provided with mealworms and housed in a 50 cm × 65 cm special container. Seven days were given to acclimate to the environment before the killing.

All related animals conformed to the guidelines for Animal Care and Use of Kunming Medical University. The protocols and procedures reached the permission of the Ethics Committee of Kunming Medical University (KMMU20180012).

### cDNA synthesis, Illumina MiSeq sequencing, and processing of data

A frog was washed with deionized water and killed. Immediately, the skin was stripped, cut into pieces, and grinded in liquid nitrogen. Total skin RNA was extracted using RNAiso (TaKaRa, Dalian, China), and mRNAs were purified using the Absolutely mRNA Purification Kit (Stratagene, Canada) according to normative protocols, then the first and second strand cDNA were synthesized via SMART techniques according to our previous studies, with the cDNA encoding frog skin bioactive precursors here screened from the library with a specific 5′ PCR primer (5′-CCAAA(G/C)ATGTTCACC(T/A)TGAAGAAA-3′) encoding the signal peptide region and 3′ PCR primer (5′-ATTCTAGAGGCCGAGGCGGCCGACATG-3′) [15]. The PCR products were recovered through a DNA gel extraction kit and then sent for Illumina MiSeq sequencing commercially. Briefly, the 50–100 ng PCR products were amplified again with KAPA HiFi Hot Start Ready Mix for three cycles, using infusion primer F (AATGATACGGCGACCACCGAGATCTACACTCTTTCCCTACACGAC GCTCTTCCGATCT-specific 5′ PCR primer) and infusion primer R (CAAGCAGAA GACGGCATACGAGATCTCAGAGTGACTGGAGTTCAGACGTGTGCTCTTCCGATCT-specific 3′ PCR primer). Amplicons were purified with Agencourt AMPure XP beads (Beckman Coulter Diagnostics, Inc., U.S.A.) according to the manufacturer’s instructions and quantitated using a Qubit 2.0 Fluorometer (Life Invitrogen, Inc., Carlsbad, CA, U.S.A.). Purified amplicons were pooled in equimolar and paired-end sequenced (2 × 300) on an Illumina MiSeq platform according to the standard protocols at Genergy Biotechnology (Shanghai, China). The adaptor sequences were removed from raw files by using Trim Galore software. Paired-end reads were assembled by FLASH-1.2.11 software with the following criteria: the overlap was between 10 and 300 bp, and the maximum of mismatch rate was 0.1. Then a file with the style of fastq was obtained and this file was transformed to the style of fasta, from which repeating sequences were removed by the Fastx-toolkit software.

### Peptide synthesis

The mature peptide of OA-GL12, ‘GLLSGINAEWPC’, was artificially synthesized and commercially provided by Wuhan Bioyeargene Biotechnology Co., Ltd. (Wuhan, China). Final purity of the synthetic peptide used for evaluating biological activities was higher than 95%.

### Cellular wound healing activity assay

Cellular wound healing activity assay was performed as previous research [8]. Briefly, human keratinocytes (HaCaT) (KCB 200442YJ) and human skin fibroblasts (HSF) (KCB 200537) were provided by Conservation Genetics CAS Kunming Cell Bank of Kunming Institute of Zoology, the Chinese Academy of Sciences, and cultured in Dulbecco’s modified Eagle’s medium (DMEM, BI, Israel) with 10% FBS (BI, Israel) and penicillin (100 units/ml)–streptomycin (100 units/ml) in a humidified atmosphere of 5% CO_2_ (37°C). The possible mycoplasma contamination was excluded by the EZ-PCR mycoplasma contamination detection kit (20-700-20, BI). Cell monolayer formation was formed by culturing the HaCaT and HSF (2.5 × 10^5^ cells/well) cells in 24-well plates for 12–24 h with serum starvation and then made to the mechanical scratch wound by a sterile pipette tip (Axygen, U.S.A.). After washing with PBS twice to detach dead cells, cells monolayer was then cultured for next periods (from 0 to 24 h) in a serum-free basal medium (500 μl) with the continued presence of OA-GL12. The same volume of DMEM without OA-GL12 was made as vehicle. In addition, DMEM-serum free (500 µl) contained mitomycin C (MMC, 10 µg/ml, Sigma–Aldrich, St. Louis, MO, U.S.A.) were set with OA-GL12 (10 pM) or not. Images of the cellular wound healing monolayers were photographed by a Primovert microscope (Zeiss, Germany) at different intervals. Cell migration activity was expressed as the percentage of the gap relative to the full area of the scratch using ImageJ software (National Institutes of Health, Bethesda, MD, U.S.A.) to evaluate the repair rate of scarification.

### Cell proliferation assays

Cells of HaCaT, HSF, human umbilical vein endothelial cells (HUVECs) (KCB 2012087YJ) and the murine macrophage cell line (RAW264.7) (KCB 200603YJ) (provided by Conservation Genetics CAS Kunming Cell Bank of Kunming Institute of Zoology, the Chinese Academy of Sciences, and the possible mycoplasma contamination was excluded by the EZ-PCR mycoplasma contamination detection kit (20-700-20, BI)) were cultured in DMEM supplemented with 10% FBS, and penicillin (100 units/ml)–streptomycin (100 units/ml) in a humidified atmosphere of 5% CO_2_ (37°C). The cells (5000 HaCaT, 10000 HSF, 5000 HUVEC, and 5000 RAW264.7 cells/well, respectively) were added into 96-well plates containing basic DMEM (90 μl) (serum free) and incubated for 2–4 h to allow the cells to adhere to well walls. Afterward, OA-GL12 (10 μl) was added to each well and incubated for 24 h at various final concentrations. The same volume of DMEM (serum free) was added as vehicle and OM-LV20 (50 nM) [8] as positive control for HaCaT. After incubation, the respective proliferative effect on HaCaT, HSF, HUVEC, and RAW264.7 cells were tested using a CellTiter 96® AQueous One Solution Assay (Promega, Madison, WI, U.S.A.) according to the manufacturer’s instructions [16].

### Transwell migration assay

Migration of HaCaT and HSF cells were tested using 24-well plates with Falcon cell culture inserts (8-μm pores; Corning, U.S.A.) as the previous study [17]. Briefly, cells were suspended in DMEM (serum free) to a concentration of 2 × 10^5^/ml and were loaded into each upper chamber with a volume of 100 µl. Then DMEM (serum free) containing different concentration gradients of OA-GL12 (600 ml) was loaded into the lower chamber. Cells were incubated at 37°C for 24 h. The nonmigratory cells on the upper surface of chambers were removed carefully. Cells were fixed with methanol for 20 min and stained with 0.1% Crystal Violet for 20 min. Counts were obtained from in five randomly selected fields under a Primovert microscope (Zeiss, Germany).

### Animal wound-healing assay

Adult male mice (22–25 g) from the same generation were purchased from Hunan SJA Laboratory Animal Co., Ltd (Certificate number 43004700043639, China). Mice were kept in individual ventilated cages (FENGSHI, China) system with laboratory animal room of Kunming Medical University. Mice were provided with essential food and water and the reverse of 12:12-h light/dark cycle. Mice were made full-thickness skin wound models after 7 days’ acclimation. Briefly, mice were anesthetized by intraperitoneal injection (i.p.) with 1% sodium pentobarbital (0.1 ml/20 g body weight). Dorsal hair were shaved by an electric clipper and sterilized with 75% alcohol. Two full-thickness skin wounds (8 × 8 mm) were surgically made on the back. After the surgery, the mice were placed in comfortable cages until they recovered from anesthesia. Nine mice were randomly divided into three groups. The right-sided wounds of the three groups were all treated with 20 µl OA-GL12. The left-sided wounds of the two groups were treated with the same volume of normal saline as vehicle, and the last group treated with 1 mg/ml KangFuXin (KFX, the ethanol extract of the *American cockroach*, Inner Mongolia Jingxin Pharmaceutical Co. Ltd, China, Z15020805) as positive control. Wounds were treated twice daily with samples and photographed at intervals of 2 days.

The condition of wounds was documented; then ImageJ software was used to estimate the wound areas (percentage of residual wound area to initial areas). Healing rate of wound (%) = [R (0) - R (2, 4, 6, 8, 10) ]/R (0) × 100%, where R (0) and R (2, 4, 6, 8, 10) denoted the remaining wound area at the same day of operation and postoperative days 2, 4, 6, 8, and 10, respectively. Wound-healing curves were constructed using GraphPad Prism software 7.0.

### Tissue preparation and histological analysis

Nine mice were made full-thickness skin wound models and randomly divided into three groups as the animal wound-healing assay. Wounds were treated with samples twice daily as the above. Mouse skin wound tissues were isolated at days 5 and 10, and analyzed with Hematoxylin and Eosin (H&E) and light microscopy. Tissue samples were fixed in 4% paraformaldehyde for 24 h, and then dehydrated and hyalinized as previous study [14]. Thick tissue sections (5 μm) were cut, deparaffinized, rehydrated, and stained by H&E for the histological analysis. The images of the slices were recorded by a Primovert microscope (Zeiss, Germany). A semi-quantitative score system was used to evaluate epidermal regeneration and granulation [18]. Evaluation of dermal and epidermal regeneration through a three-point scale: (1, little regeneration; 2, moderate regeneration; 3, complete regeneration). To evaluate granulation tissue formation using a four-point scale: (1, thin granulation layer; 2, moderate granulation layer; 3, thick granulation layer; 4, very thick granulation layer). The situation of wound was evaluated by IPLab imaging software (BD Biosciences, Bedford, MA, U.S.A.). Neo-epithelium breadth and distances of wound site were measured, then the percentage of re-epithelialization (%) = [(distance covered by neo-epithelium)/(distance between wound site) × 100] was analyzed.

### Inflammatory assay

RAW264.7 cells were cultured in DMEM (BI, Israel) supplemented with 10% (v/v) FBS Hyclone) and antibiotics (100 units/ml penicillin and 100 units/ml streptomycin) at 37°C in a humidified atmosphere of 5% CO_2_. As for the immunomodulatory activity of samples, tumor necrosis factor (TNF) and transforming growth factor β1 (TGF-β1) were tested referring to previous researches [15,19]. In short, the cells (1 × 10^5^/well, respectively) were plated in 96-well plates with serum-free medium (90 μl). After 3 h, for cells to adhere, samples (10 μl) were added into the wells reaching different concentrations, while control group was added with same volume of serum-free medium. After incubation for 12 h, supernatants were collected to detect the excretion of TNF and TGF-β1 by an ELISA kit (NEOBIOSCIENCE, China) following the manufacturer’s protocols.

### Assay of epidermal growth factor receptor inhibitor on the cellular wound healing activity of OA-GL12

HaCaT cells were cultured in DMEM with 10% FBS and penicillin–streptomycin in a humidified atmosphere of 5% CO_2_ (37°C). HaCaT cells (2.5 × 10^5^ cells/well) were added in 24-well plates for 12–24 h with serum starvation and then made to the mechanical scratch wound by a sterile pipette tip. After washing, cells monolayer was then cultured for next periods (from 0 to 24 h) in a serum-free basal medium (500 μl) with the continued presence of Gefitinib (1 μM, HY-50895, MedChemExpress, China), OA-GL12 (10 pM) and OA-GL12 (10 pM) with Gefitinib (1 μM) [20]. The same volume of DMEM was made as a vehicle. Images were photographed at different intervals. Cell healing activity was expressed as the percentage of the gap relative to the full area of the scratch to evaluate the repair rate of scratch.

### Hemolytic activity assay

Hemolytic activity assay was performed according to previous research [21]. Briefly, erythrocytes were triply and softly washed with 0.9% normal saline. Then cells were incubated with various concentrations of OA-GL12 for 30 min (37°C), while 0.1% Triton X-100 as maximum hemolysis. The absorbance of supernatant was read at 540 nm after centrifugation with 3000×***g*** for 4 min at room temperature. The numeric data of hemolytic activity (%) was calculated by [(A_sample_) × 100]/A_triton x-100_.

### Acute toxicity assay

Two phases of acute toxicity were performed as previous study [22]. Lethal concentration of OA-GL12 was tested at 5, 10, 25, and 50 μmol/kg by i.p. in mice, while using saline as negative control. The mortalities, toxic effects, and behavioral changes were observed and recorded for 2 weeks.

### Free radicles scavenging activities

2,2′-azino-bis (3-ethylbenzothiazoline-6-sulphonic acid) (ABTS) and 2,2-diphenyl-1-picrythydrazyl (DPPH)) scavenging activities of OA-GL12 were assessed referring to previous studies [23,24]. Briefly, a stock solution of ABTS radical was prepared by incubating 2.8 mM potassium persulphate (Sigma–Aldrich, St. Louis, MO, U.S.A.) with 7 mM ABTS in water for no less than 6 h in the dark, and used immediately. The stock solution was diluted 50-fold with double distilled water. Dissolved samples with ultrapure water were added to the diluted stock solution, while the same volume of solvent was used as the blank, and Vitamin C as the positive control. The reaction was in the dark for 30 min. The decrease in absorbance at 415 nm showed antioxidant activity of the samples. Meanwhile, the assay mixture contained 190 μl of 5 × 10^−5^ M DPPH radical (Sigma–Aldrich, St. Louis, MO, U.S.A.) dissolved in ethanol and 10 μl of sample solution. The mixture was then incubated in a 96-well microtiter plates at room temperature for 30 min. Subsequently, the absorbance was measured at 517 nm. The free radical scavenging activity (%) was calculated by (A_blank_ − A_sample_) × 100/A_blank_.

### Antimicrobial activity assay

The antimicrobial activity of OA-GL12 was performed based on our previous research [21]. Briefly, fungal strain *Candida albicans* (ATCC 14053), Gram-positive bacterial strains *Staphylococcus epidermidis* (ATCC 12228), *Staphylococcus haemolyticus* (ATCC 29970), and *Bacillus subtilis* (ATCC 19659), Gram-negative bacterial strains *Escherichia coli* (ATCC 25922) were obtained from the Kunming Medical University. The microbes were cultivated in LB broth to OD_600_ = 0.8. A 10-μl aliquot of the bacteria was collected and added to fresh LB (10 ml) broth with 1% Type I agar (Sigma–Aldrich, St. Louis, MO, U.S.A.), and then poured into a 90-mm Petri dish. A micro hole was made after the agar was solidified, and a 7-μl aliquot of OA-GL12 (1 mM) was added to the hole. Petri dish was placed for 16–18 h at 37°C. As the symbol of antimicrobial activity, a clear zone appeared on the surface of the agar just like ampicillin (1 mg/ml), positive control, which represented the antimicrobial ability of OA-GL12.

### Stability analysis of OA-GL12

The stability of OA-GL12 at 4 and 37°C was performed as previous study [13]. Briefly, tubes containing OA-GL12 (10 μg/ml dissolved in deionized water) were stored at 4 and 37°C for appointed days after centrifugation (12000×***g***, 20 min), supernatants were removed and analyzed by C18 RP-HPLC (Hypersil BDS C18, 4.0 × 300 mm, Elite, China). The initial and residual levels of OA-GL12 were quantitated from peak areas.

### Statistical analysis

Data were depicted as mean ± S.D. Statistical significance of differences was determined by *t* test or nonparametric test (Mann–Whitney test) when compared in two groups; one-way ANOVA or nonparametric test (Kruskal–Wallis test) analysis when compared in more than two groups. Normality was tested by Shapiro–Wilk test, while homogeneity of S.D. was determined by F test, Brown–Forsythe and Bartlett’s test. Statistical analysis was performed by GraphPad Prism 7.0 (GraphPad software, San Diego, U.S.A.) and considered significant at *P*<0.05.

## Results

### The discovery of OA-GL12

**Figure 1:**
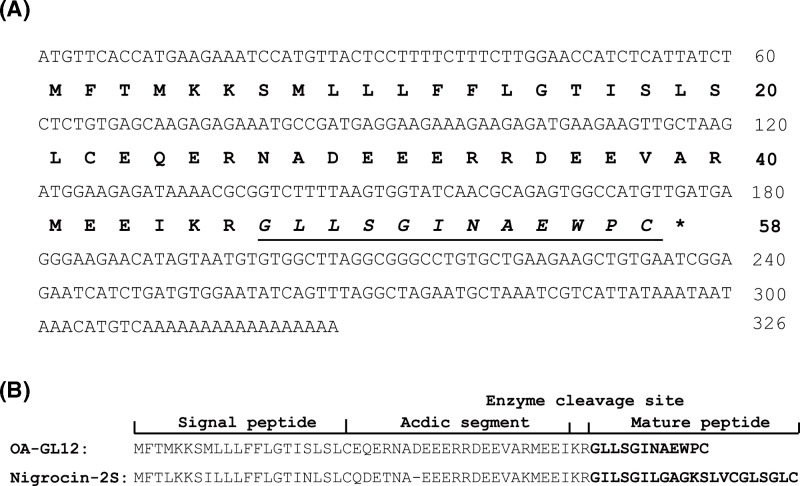
Sequence of cDNA encoding OA-GL12 cDNA synthesis, Illumina MiSeq sequencing, processing of data, and peptide artificial synthesis. (**A**) The sequence of cDNA encoding OA-GL12. The precursor of OA-GL12 was composed of 58 animo acid residues and encoded by an mRNA composed of 326 bp. The mature sequence of OA-GL12 was assumed as ‘GLLSGINAEWPC’, which is shown in underline and italics. (**B**) The sequence alignments between OA-GL12 and Nigrocin-2S. Precursors of OA-GL12 and Nigrocin-2S shared sequence similarity, but no obvious similarity on mature peptides was observed. Meanwhile, differing from OA-GLA12, Nigrocin-2S was composed of an intramolecular disulphide bond located at C-terminus. The overall structures of these peptides shared highly conserved motif, which are divided into four parts: signal peptide, acidic segment, enzyme cleavage site, and the highly variable mature peptide.

In this research, we obtained a cDNA sequence encoding a peptide precursor from the skin of *O. andersonii*. As shown in Figure 1A, this precursor of 58 animo acid residues in length was encoded by an mRNA composed of 326 bp. By BLASTp in NCBI, we found this precursor showed high similarity to the frog antimicrobial peptides, such as Nigrocin-2S (Figure 1B). The overall structures of these peptides shared highly conserved motif, which are divided into four parts: signal peptide, acidic segment, enzyme cleavage site, and the highly variable mature peptide, thus, the mature peptide sequence of this precursor was assumed as ‘GLLSGINAEWPC’. Of note, this mature peptide is a linear motif, which is quite different from the cyclic motif of Nigrocin-2S, moreover, it showed no obvious sequence similarity with other bioactive frog peptides and thus was considered to be novel and named as OA-GL12 according to our previous reports (OA: abbreviation of species, GL: two initial amino acids, 12: peptide length) [13,14].

### OA-GL12 promoted HaCaT cell scratch healing

**Figure 2:**
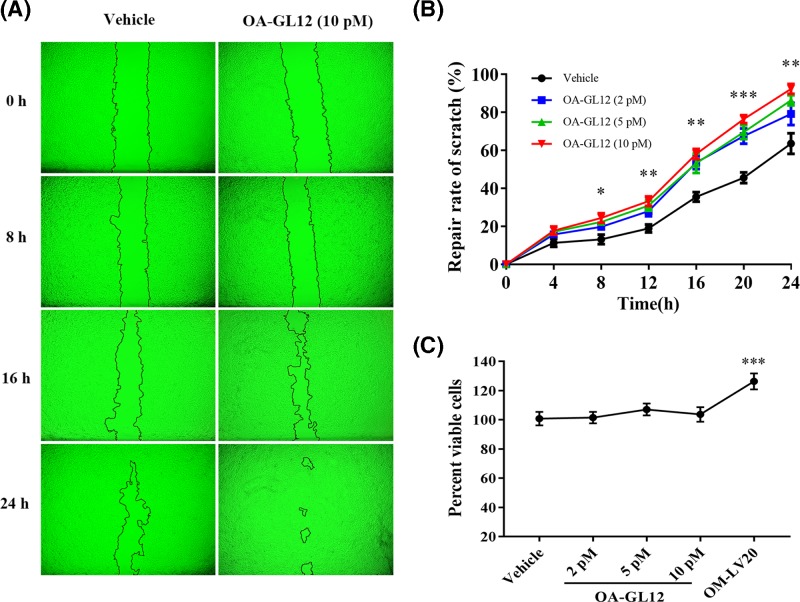
Effect of OA-GL12 on the repair rate of HaCaT cells scratches HaCaT cells (2.5 × 10^5^ /well) were cultured in 24-well plates for 12–24 h with serum starvation and then made to the mechanical scratch wound by a sterile pipette tip. After washed, cells were cultured for next periods (from 0 to 24 h) in a serum-free basal medium with the continued presence of OA-GL12. The same volume of DMEM (serum free) was added as vehicle and OM-LV20 (50 nM) as positive control. (**A**). OA-GL12 (10 pM) showed prominent wound-healing capacity on HaCaT cells. (**B**). OA-GL12 illustrated time- and concentration- dependent wound-healing capacity on HaCaT cells. Contain cells were added into 96-well plates with basic DMEM (90 μl) without 10% FBS and incubated for 2-4 h. Afterward, OA-GL12 (10 μl) was added to each well and incubated for 24 h at various final concentrations then tested using a CellTiter 96® AQueous One Solution Assay. (**C**). OM-LV20 (50 nM) showed proliferative effect, but OA-GL12 did not show on HaCaT cells from 2 to 10 pM. Data were presented as mean ± S.D., *n*=9. *P<0.05, **P<0.01, and ***P<0.001 (one-way ANOVA or nonparametric test).

Keratinocytes play crucial roles in the repair of cutaneous lesion. They migrate immediately to damaged areas and proliferate to activate re-epithelialization of the wound incision. In the present study, we performed cell scratch assays to observe the healing effect of OA-GL12 on HaCaT cells from 0 to 24 h at an interval of 4 h. As illustrated in Figure 2A, OA-GL12 (10 pM) showed outstanding promoting cell scratch-healing capacity and the scratch region of OA-GL12-treated appeared narrower than control from 0 to 24 h. Notably, OA-GL12 exerted scratch-healing accelerating activity in both time- and concentration-dependent manner (Figure 2B). OA-GL12-treated group (10 pM) showed significant scratch-healing rates of 24.4 ± 2.51% at 8 h, 58.07 ± 2.67% at 16 h, 92.31 ± 2.61% at 24 h, respectively, while the vehicle group healing rates were 13.1 ± 2.52% at 8 h, 35.46 ± 2.47% at 16 h, 63.53 ± 5.41% at 24 h, respectively. These results showed that OA-GL12 effectively promoted cell scratch healing in both time- and concentration-dependent manner (2–10 pM, Figure 2A,B).

Considering that the proliferation of HaCaT cells also dominantly takes part in proliferation phase of wound healing, therefore, an MTS assay was performed to detect the effects of OA-GL12 on HaCaT cell proliferation. As shown in Figure 2C, OA-GL12 did not obviously stimulate the proliferation of HaCaT cells at concentrations of 2, 5, and 10 pM when compared with vehicle and OM-LV20.

In order to rule out the effect of proliferation, we set MMC groups with OA-GL12 (10 pM) or not. The migratory cells in each group were observed by microscopy (Figure 4A). As shown in Figure 4C, the healing rate of the HaCaT cells in MMC group (26.66 ± 4.64%) decreased sharply when compared with vehicle (53.39 ± 10.11%), while it significantly increased once added with OA-GL12 (10 pM) (63.45 ± 7.06%). Regardless of whether the proliferation of HaCaT cells was inhibited or not, OA-GL12 did show wound healing activity on HaCaT cell scratches. Comprehensive consideration of the above results, we assumed that OA-GL12 promoted HaCaT cell scratch healing mainly by stimulating cell migration, rather than cell proliferation (Figures 2 and 4A,C).

### OA-GL12 promoted HSF cell scratch healing

**Figure 3:**
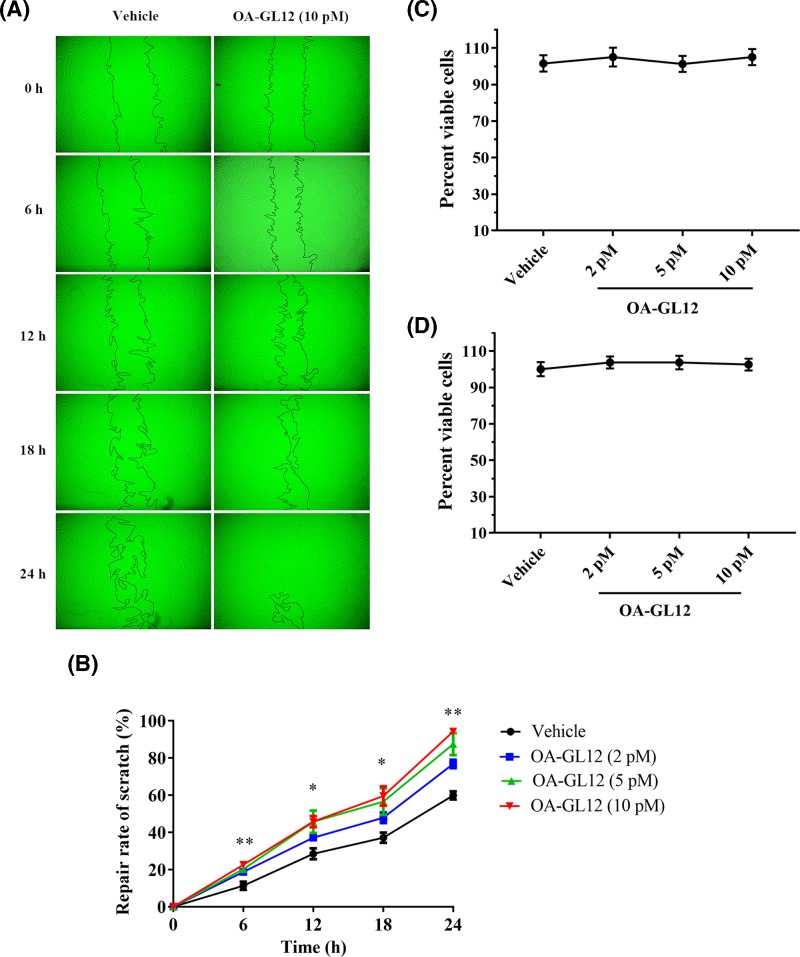
Effect of OA-GL12 on the repair rate of HSF cell scratches HSF cells (2.5 × 10^5^ /well) were cultured in 24-well plates for 12–24 h and then made to the mechanical scratch wound by a sterile pipette tip. After washing, cells were cultured for next periods in a serum-free basal medium (500 μl) with the continued presence of OA-GL12. The same volume of DMEM (serum free) was added as vehicle. (**A**) OA-GL12 (10 pM) illustrated distinct HSF cells scratch-healing activity. (**B**) OA-GL12 illustrated time- and concentration-dependent HSF cells scratch-healing activity. Contain cells were added into 96-well plates with basic DMEM (90 μl) without 10% FBS and incubated for 2–4 h. Afterward, OA-GL12 (10 μl) was added to each well and incubated for 24 h at various final concentrations then tested using a CellTiter 96® AQueous One Solution Assay. (**C**,**D**). OA-GL12 showed no impact on the proliferation of HSF and HUVEC cells from 2 pM to 10 nM. Data were presented as mean ± S.D., *n*=9. *P<0.05 and **P<0.01 (one-way ANOVA or nonparametric test).

**Figure 4:**
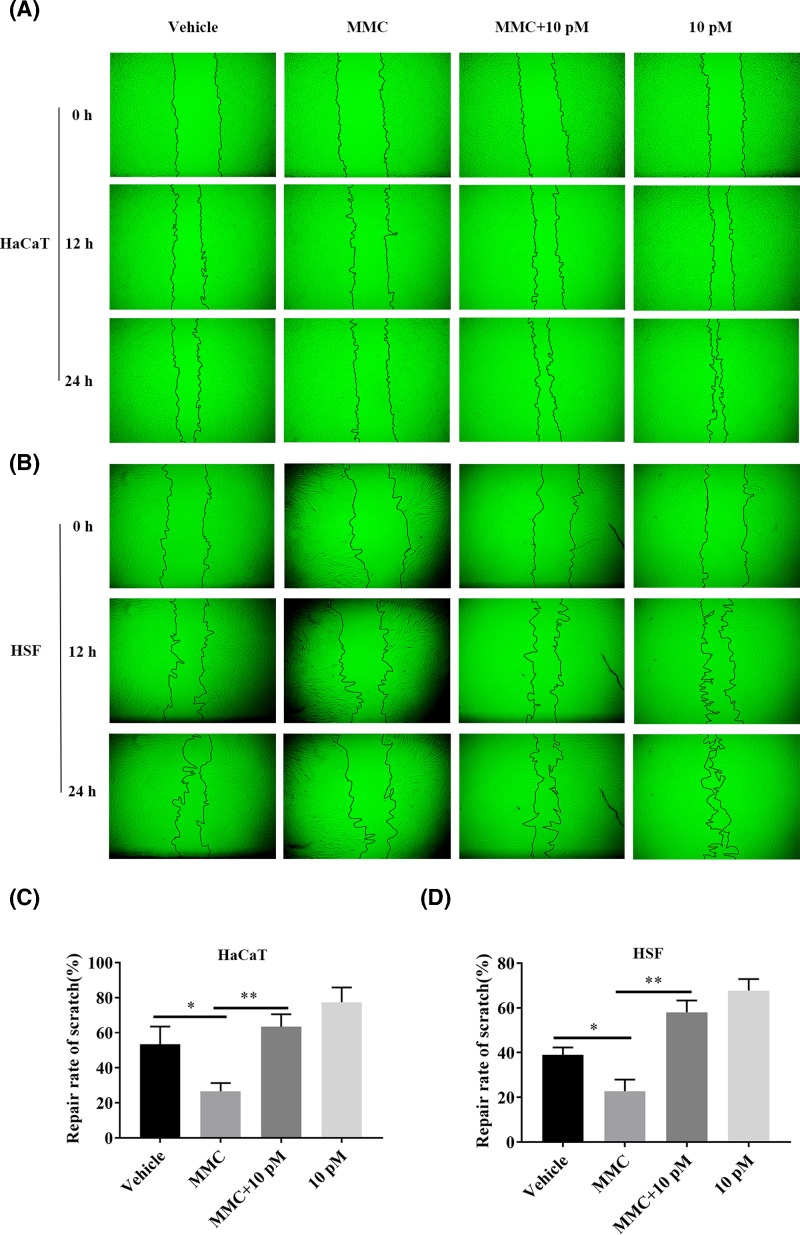
Effect of OA-GL12 on the repair rate of HaCaT and HSF cell scratches with MMC or not Cells (2.5 × 10^5^ /well) were cultured for 12–24 h and then made to the mechanical scratch wound. After washing, cells were cultured for next periods in a serum-free basal medium (500 μl) with the continued presence of OA-GL12 (10 pM) with MMC or not. The same volume of DMEM (serum free) was added as vehicle, and the same volume of DMEM (serum free) with MMC was added as MMC. (**A**,**B**). The status of cell growth in each group were observed by microscope in 24 h. OA-GL12 (10 pM) illustrated distinct HaCaT and HSF cells scratch-healing activity with MMC or not. (**C**,**D**). The repair rate of each group at 24 h. OA-GL12 still showed strong scratch-healing activity on HaCaT and HSF cells with MMC inhibiting cells proliferation. Data were presented as mean ± S.D., *n*=9. *P<0.05 and **P<0.01: two groups connected by the horizontal line were tested by *t* test or nonparametric test.

Fibroblasts and umbilical vein endothelial cells are parts of the formation of granulation tissue. The effect of OA-GL12 on the elevation of scratch closure capacity on HSF cells was also tested in this research. As illustrated in Figure 3A, OA-GL12 (10 pM) showed outstanding scratch closure capacity by migrating inwardly and covering bigger areas of the scratch than the vehicle. The results of Figure 3B demonstrated that OA-GL12 enhanced the closure of HSF cell scratches in both time- and concentration-dependent manner. OA-GL12 (10 pM) displayed significant scratch closure healing rates of 45.76 ± 3.03% at 12 h, 94.23 ± 1.28% at 24 h, respectively, while the negative control showed rates of 28.48 ± 2.90% at 12 h, 59.9 ± 2.22% at 24 h, respectively (Figure 3B). In addition, OA-GL12 (10 pM) (58.02 ± 5.31%) also showed strong scratch-healing activity when proliferation was inhibited by MMC, however, the scratch healing of MMC reduced (22.78 ± 5.22%) when compared with vehicle (38.96 ± 3.34%) (Figure 4B,D). As displayed in Figure 3C,D, the MTS assay results suggested OA-GL12 had no contribution to HSF or HUVEC cell proliferation. In conclusion, OA-GL12 induced the healing of HSF cell scratches closure mainly by inducing cell migration rather than proliferation (Figures 3 and 4B,D).

### OA-GL12 promoted HaCaT and HSF cells migration

**Figure 5:**
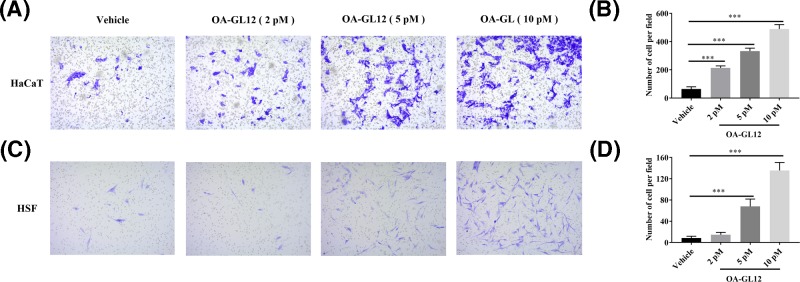
OA-GL12 promoted cells migration in a concentration dependent manner HaCaT and HSF cells were trypsin digested, counted, washed, and adjusted to 2 × 10^5^ cells/ml. Cells (100 μl) were added into upper chambers of transwell, while bottom chambers contained DMEM (serum free) with OA-GL12 at 0, 2, 5, or 10 pM for 24 h to induce the cell migration. Cells were fixed with methanol and stained with 0.1% Crystal Violet. Counts were obtained from in five randomly selected fields. (**A**,**C**) The cells travelling to the bottom chamber were observed by microscopy. (**B**,**D**). The number of migratory cells increased significantly in concentration-dependent manner when compared with vehicle. Data were presented as mean ± S.D., *n*=9. ***P<0.001: two groups connected by the horizontal line were tested by *t* test or nonparametric test.

To verify the effect of OA-GL12 on HaCaT and HSF cells, migration was tested using 24-well plates with Falcon cell culture inserts (8-μm pores; Corning, U.S.A.) [17]. Cells were induced with OA-GL12 (0, 2, 5, or 10 pM) for 24 h. Subsequently, the cells travelling to the bottom chamber were observed by microscopy (Figure 5A,C). Results demonstrated that OA-GL12 (2, 5, or 10 pM) significantly increased numbers of HaCaT and HSF cells migration when compared with vehicle (0 pM) at 24 h (Figure 5B,D), which were consistent with the above results. These observations suggested that OA-GL12 promoted cells migration in a concentration-dependent manner (Figure 5). The activity of stimulating HaCaT cell migration might be due to epidermal growth factor receptor (EGFR) transactivation and signaling intermediates extracellular signal-regulated kinase (ERK) 1/2 (ERK1/2) and Smad2, additionally, some reports also suggested that the phosphatidylinositol 3-kinase/protein kinase B (PI3K/AKT) and c-Jun N-terminal kinase (JNK) pathways are two potential signaling pathways related to cell migration [20,25–28]. As for HSF cells, nuclear factor κB (NF-κB) and ERK pathways might contribute to the capacity of migration [29].

### OA-GL12 promoted wound healing on full-thickness skin wound mice

**Figure 6:**
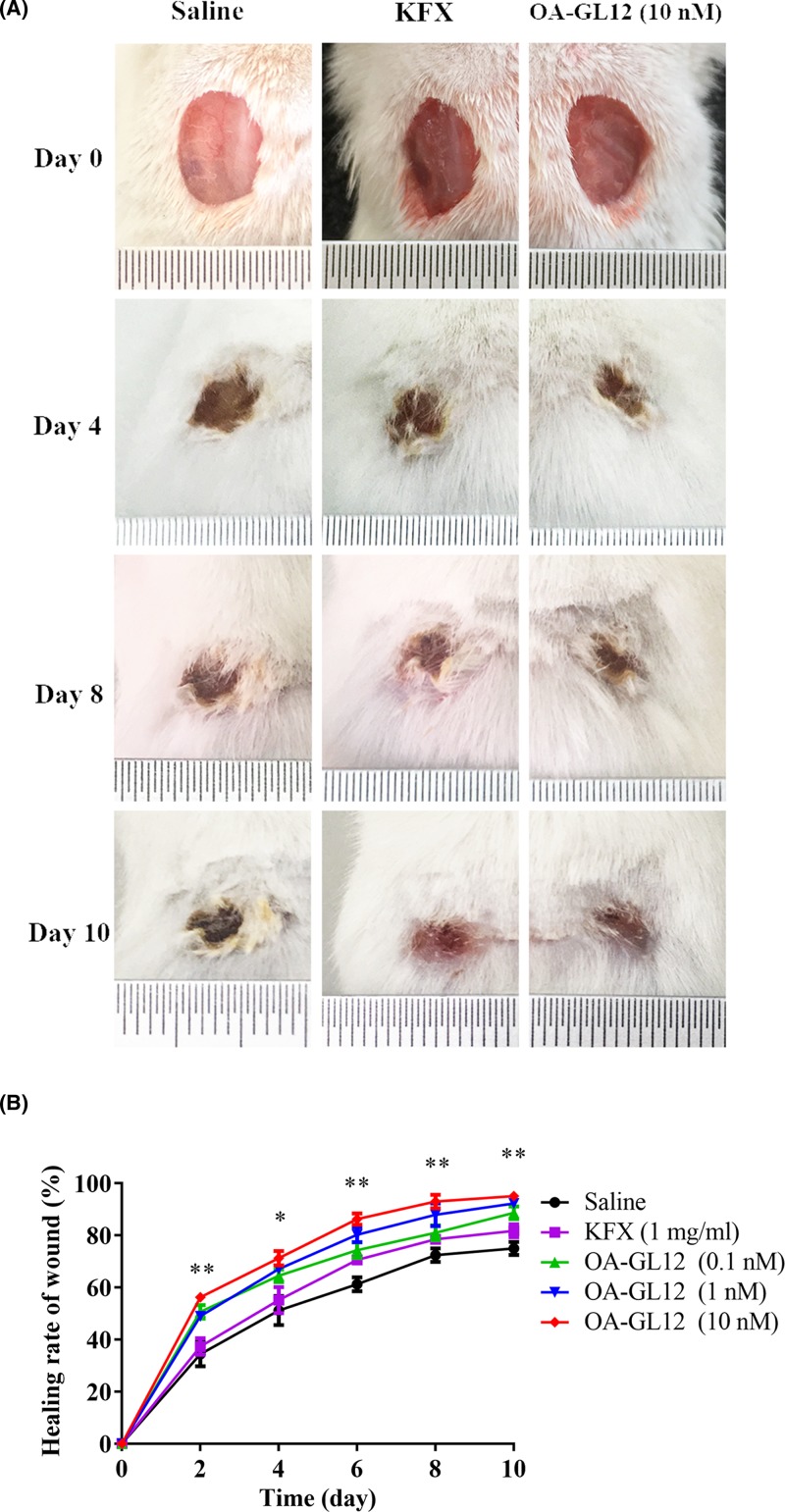
Effect of OA-GL12 on full-thickness skin excisional wound healing mice models Wounds (8 × 8 mm) were made on the back of mice, which were treated twice on one day with 20 μl of saline (negative control), KFX (1 mg/ml, positive control), and OA-GL12 (0.1, 1, 10 nM), respectively. Images of representative mice on postoperation days 0, 4, 8, and 10 were exhibited. (**A**) Gross appearance of wound on days 0, 4, 8, and 10. (**B**) Promoting wound healing activity of OA-GL12 showed time- and dose-dependent activity on the mice models. Wound closure was measured by morphometrical analysis (ImageJ, NIH). Data were presented as mean ± S.D. *n* (the Saline group)=6, *n*(other groups)=3, respectively. *P<0.05 and **P<0.01 (one-way ANOVA or nonparametric test).

OA-GL12 showed effective scratch accelerating activity *in vitro*, accordingly, wound-healing activity was evaluated *in vivo* by using full-thickness skin excisional wound healing mouse model. Wounds of mouse model were respectively applied with OA-GL12 (0.1, 1, and 10 nM, 20 μl), KFX (1 mg/ml, 20 μl, positive control), and 0.9% saline (negative control, 20 μl), twice daily. At postoperative day 10, the wound healing of the mouse treated with OA-GL12 (10 pM) were better than the mouse treated with saline or KFX (Figure 6A). As illustrated in Figure 6B, wound healing rate of OA-GL12 (10 nM) increased from 56.18 ± 0.53 to 95.03 ± 1.90%, while that of KFX-treated mice increased from 37.3 ± 3.19 to 81.7 ± 2.5%, saline-treated mouse increased from 34.34 ± 4.73 to 74.95 ± 2.5%, respectively. In addition, OA-GL12 (0.1 nM) shared parallel effect with the KFX on skin wound repair *in vivo* (Figure 6B). No adverse effects on mouse body weight, general health, or behavior were found during the whole OA-GL12 treatment (data not shown).

**Figure 7:**
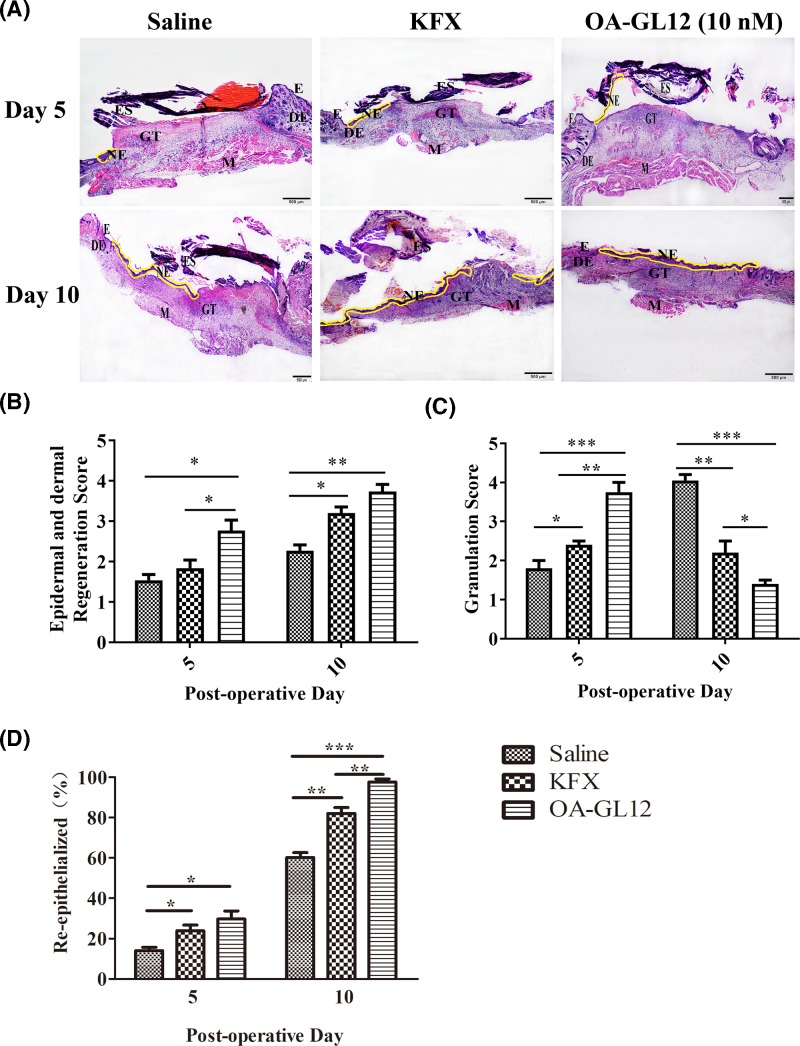
Histopathological examination of mice wound treated with OA-GL12 (10 nM) Wounds were treated with samples twice daily as above. Mouse skin wound tissues were isolated at days 5 and 10, tissue samples were fixed in 4% paraformaldehyde and dehydrated and hyalinized. Thick tissue sections (5 μm) were cut, deparaffinized, rehydrated, and stained by H&E for histopathological examination. The images of the slices were recorded by a Primovert microscope. (**A**) Histopathological examination of saline, KFX, and OA-GL12-treated (10 nM) full-thickness excisional wounds stained with H&E (NE, neo-epidermal; GT, granulation tissue; DE, dermis; E, epidermis; ES, eschar; M, muscle; Scale bar: 500 μm). Neo-epithelium marked by yellow line was much longer at the same magnitude of enlargement in OA-GL12-treated mice than that in controls. (**B**,**C**) Histological scores of epidermis and dermis regeneration and granulation. (**D**) Percent of re-epithelialization. Data were presented as mean ± S.D., *n*=8. *P<0.05, **P<0.01, and ***P<0.001: two groups connected by the horizontal line were tested by *t* test or nonparametric test.

In a parallel experiment, mice were killed for histological analysis at postoperative days 5, and 10, and the effects of OA-GL12 on wound healing *in vivo* were further investigated. Histological analysis indicated that mice treated with OA-GL12 (10 nM) displayed prominently accelerating regeneration of neo-epidermis (neo-epithelial tongue) and restoration of dermis (granulation tissue) in the wound compared with the saline or KFX group. At day 5, the saline control section stayed in inflammation phase (severest edema, hemorrhage), while OA-GL12-treated group showed best re-epithelialization and best-formed granulation tissue amongst three groups. At day 10, epidermal regeneration and reconstruction of the dermis were complete in the OA-GL12 group, which were similar to that in normal mice (Figure 7A). Histological evaluation of mice skin tissue sections stained with H&E was also carried out. As illustrated in Figure 7B–D, results demonstrated that OA-GL12 promoted regeneration of neo-epidermal tissues and formation of dermal in the wound site. On postoperative day 10, neo-epithelial tongue was completely covered the whole wounded area and granulation tissue in the OA-GL12-treated mice (re-epithelialization 97.67 ± 1.45%). In contrast, only little partial neo-epithelial tongue was found in the saline (re-epithelialization 60.21 ± 2.40%) and most partial neo-epithelial tongue in the KFX (re-epithelialization 82.08 ± 2.85%) on same day (Figure 7A,B,D). In conclusion, histological analysis revealed that mice treated with OA-GL12 (10 nM) showed significant increase in the regeneration of epidermis and dermis, better granulation tissue contraction, and high re-epithelialization, in comparison with saline and KFX group (Figure 7).

### Effect of OA-GL12 on secretions of TNF and TGF-β1

**Figure 8:**
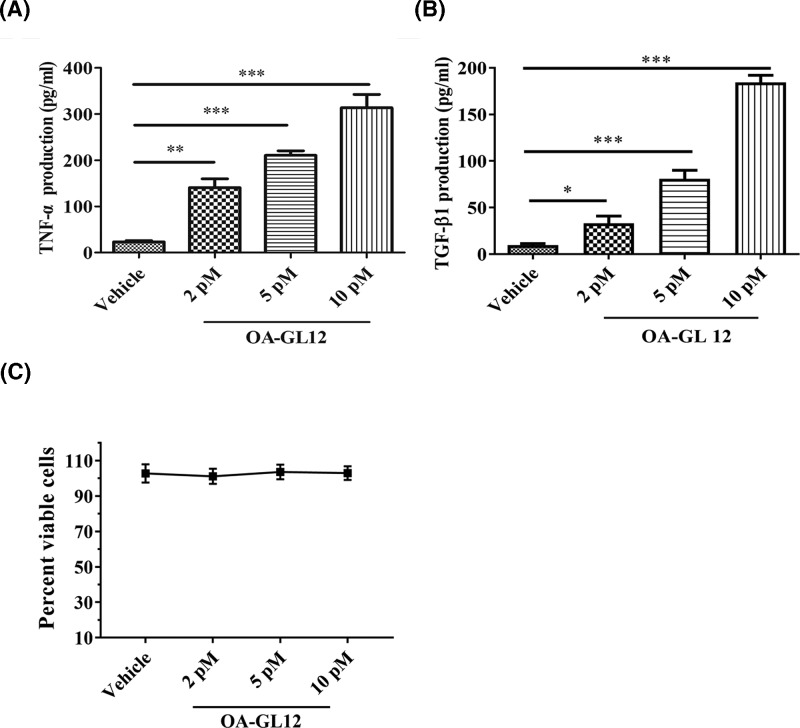
Effect of OA-GL12 on the secretion of TNF and TGF-β1 in macrophage cell RAW264.7 RAW264.7 (1 × 10^5^/well) were added in 96-well plates with serum-free medium (90 μl) respectively for 3 h. Then samples (10 μl) were added into each well reaching different concentrations, while control group contained same volume of serum-free medium. After incubation for 12 h, supernatants were collected to detect the excretion of TNF and TGF-β1 by ELISA kit. (**A**,**B**) OA-GL12 (2, 5, 10 pM) induced TNF and TGF-β1 secretion in a concentration-dependent manner. (**C**) Effect of OA-GL12 on RAW264.7 cell proliferation. The values treated with three different concentrations of OA-GL12 (2, 5, 10 pM) showed no significant difference from that of vehicle. Data were presented as mean ± S.D., *n*=6. *P<0.05, **P<0.01, and ***P<0.001: two groups connected by the horizontal line were tested by *t* test or nonparametric test.

Macrophages participate in each stage of wound healing including the inflammation, cell proliferation, and tissue reconstruction [30]. Recent researches showed that TNF and TGF-β1, mostly secreted by macrophages, have been the focus, owing to their important roles in wound healing process [31–33]. In the early phase of inflammation during wound healing, TNF can be up-regulated timely which recruits various inflammatory cells (macrophages, et al.) to secrete more TGF-β1 to wound sites for further wound healing by its chemotactic functions. Moreover, TGF-β1 can generate the complicated processes of cellular proliferation, migration, and granulation tissue regeneration [34]. To explore the possible molecular mechanism of OA-GL12 on wound healing, the secretion of TNF and TGF-β1, induced by the application of OA-GL12 on RAW264.7 cells, were tested by ELISA method. As illustrated in Figure 8A, OA-GL12 improved the secretion of TNF and TGF-β1 in a concentration-dependent manner. After incubation with OA-GL12 at different concentrations (2, 5, and 10 pM) for 12 h, the TNF concentration in the supernatant increased from 23.0 ± 3.06 to 140.3 ± 19.24, 210.3 ± 9.87, 313.3 ± 29.06 pg/ml, respectively. In addition, TGF-β1 secretion also increased from 9.74 ± 1.74 to 33.1 ± 7.74, 80.74 ± 9.26, 184.4 ± 7.65 pg/ml, respectively, after incubation with different concentration of OA-GL12 for 12 h (Figure 8B). As illustrated in Figure 8C, result demonstrated that OA-GL12 had no contribution to RAW264.7 cell proliferation, which suggested that the secretion of pro-inflammatory cytokine in RAW264.7 cells was not induced by cell proliferation.

### EGFR was associated with scratch healing of OA-GL12 on HaCaT cells

**Figure 9:**
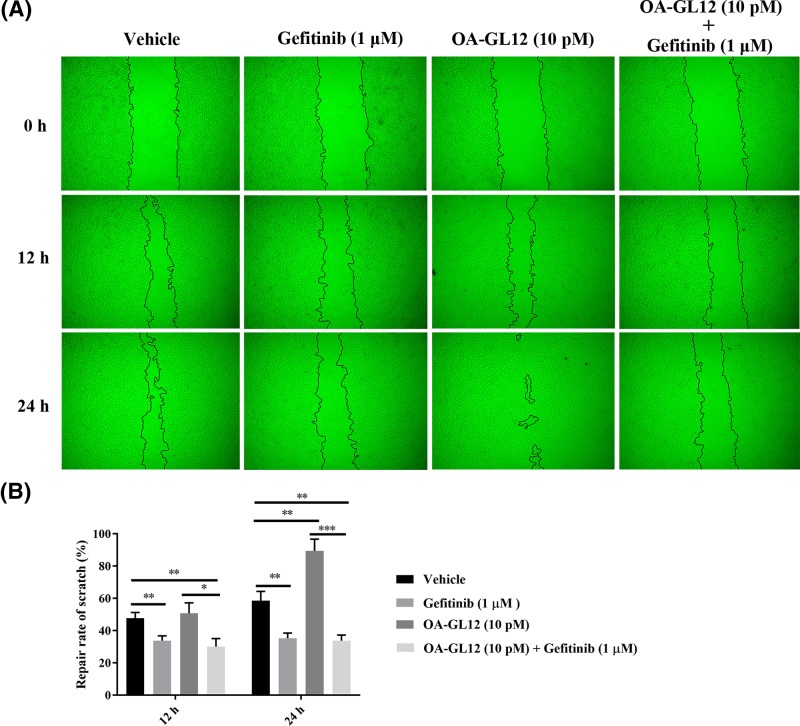
Effect of EGFR inhibitor on the prohealing activities of OA-GL12 on HaCaT cells HaCaT cells (2.5 × 10^5^ /well) were cultured for 12–24 h, then made the mechanical scratch wound. After washing, cells were cultured for next periods (from 0 to 24 h) in a serum-free basal medium with the continued presence of Gefitinib (1 μM), OA-GL12 (10 pM), and OA-GL12 (10 pM) with Gefitinib (1 μM). The same volume of DMEM (serum free) was added as vehicle. (**A**) OA-GL12 (10 pM) showed prominent wound scratch-healing capacity on HaCaT cells but not when added Gefitinib (1 μM). (**B**) Repair rates of scratch in the Gefitinib (1 μM) group were lower when compared with vehicle, while OA-GL12 (10 pM) illustrated significant difference on the repair rates of scratch. Data were presented as mean ± S.D., *n*=9. *P<0.05, **P<0.01, and ***P<0.001: two groups connected by the horizontal line were tested by *t* test or nonparametric test.

EGFR signaling pathway is one of the most important pathways that stimulate epidermal cell growth. In the present study, we performed cell scratch assays to further explore the possible healing effect mechanism of OA-GL12. As reported in previous researches, Gefitinib (1 μM) showed no obvious cytotoxicity on HaCaT cells after the treatment for 24 h (data not shown). As illustrated in Figure 9A, OA-GL12 (10 pM) showed strong activity on promoting cell scratch-healing and the scratch region appeared narrower than other groups from 0 to 24 h. However, the scratch regions in groups treated with Gefitinib (1 μM) were wider when compared with vehicle. Notably, OA-GL12 did not exert scratch-healing accelerating activity when treated with Gefitinib (Figure 9B). OA-GL12-treated group (10 pM) showed significant scratch-healing rates from 50.67 ± 6.51 to 89.33 ± 7.37%, while the group OA-GL12 with Gefitinib from 30.51 ± 5.02 to 33.67 ± 3.51%, respectively.

### OA-GL12 showed no hemolytic activity *in vitro* and no acute toxicity *in vivo*

In order to test the safety of potential clinical application as a wound-healing candidate, we also performed the hemolysis and acute toxicity test of OA-GL12. As a result of hemolytic activity, OA-GL12 showed negligible hemolytic activity on human erythrocytes *in vitro* (Table 1). With regard to acute toxicity, we also investigated mice model with different range i.p. injection of OA-GL12 at different concentrations and found no death and other abnormal behaviors, such as tremor, retardation, irritability, piloerection, and stiffness of tail in 2 weeks (Table 2). The above results indicated the potential safety of OA-GL12 for therapeutic application.

**Table 1:** Hemolytic activity of OA-GL12 in human erythrocytes

Group	Hemolytic ratio (%)
Triton X-100	100
Saline	8.7 ± 0.56
0.1nM (OA-GL12)	8.9 ± 0.43
1 nM (OA-GL12)	9.8 ± 0.45
10 nM (OA-GL12)	9.9 ± 0.75

Data were presented as mean ± S.D., *n*=6.

**Table 2:** Mouse mortality at diverse injection doses of OA-GL12

Group	Dosage	Number		Mortality (%)
	(μmol/kg)	Male	Female	
Control	50	3	3	0
OA-GL12	5	3	3	0
	10	3	3	0
	25	3	3	0
	50	3	3	0

Data were presented as mean ± S.D., *n*=6.

### OA-GL12 showed free radicals scavenging activity

**Figure 10:**
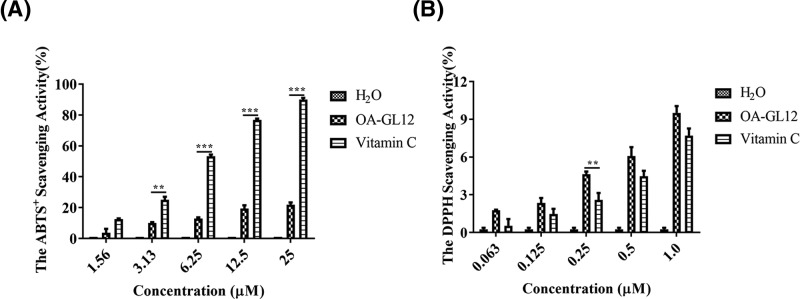
Free radical scavenging activities of OA-GL12 Dissolved samples with ultrapure water were added to the diluted stock solution (ABTS radical), while the same volume of solvent was used as the blank, and Vitamin C as positive control. After 30 min in the dark, the mixture was measured at 415 nm. (**A**) ABTS^+^ free radical scavenging activity of OA-GL12 was obvious in concentration-dependent manner. The assay mixture contained 190 μl of 5 × 10^−5^ M DPPH radical dissolved in ethanol and 10 μl of sample solution. After 30 min in the dark, the mixture was measured at 517 nm. (**B**) OA-GL12 showed DPPH scavenging activity in a concentration-dependent manner. Data were presented as mean ± S.D., *n*=3. ‘H_2_O’ indicated negative control; ‘Vitamin C’ indicated positive control. Column statistics was performed between OA-GL12 and H_2_O. **P<0.01 and ***P<0.001: *t* test or nonparametric test was performed between OA-GL12 and Vitamin C.

As shown in Figure 10A, OA-GL12 displayed obvious but weaker (compared with positive control Vitamin C) ABTS^+^ free radical scavenging activity at the concentrations ranging from 1.56 to 25 μM. As illustrated in Figure 10B, however, OA-GL12 showed stronger (compared with positive control Vitamin C) DPPH scavenging activity in a concentration-dependent manner. At maximum concentration of 1 μM, OA-GL12 scavenged 9.50 ± 0.54%, while Vitamin C scavenged only 7.71 ± 0.56% of DPPH scavenging. Even at the minimum concentration of 0.063 μM, the antioxidant activity of OA-GL12 still showed obvious DPPH scavenging activity, while Vitamin C was nearly inactive. These results indicated that the activity of OA-GL12 was more powerful than Vitamin C in DPPH scavenging, but weaker in ABTS^+^ scavenging.

### OA-GL12 showed no antimicrobial activity

Because the precursor of OA-GL12 and antimicrobial peptide Nigrocin-2S showed sequence similarity, we next determined the potency of OA-GL12 against fungi and bacteria. As listed in Table 3, OA-GL12 showed no direct killing effect on fungal strain *C. albicans*, Gram-positive bacterial strains *S. epidermidis, S. haemolyticus*, and *B. subtilis*, Gram-negative bacterial strain *E. coli*, even at a maximum concentration of 1 mM.

**Table 3:** Antimicrobial activity of OA-GL12

Microorganisms	Antimicrobial activity	
	Amp (1 mg/ml)	OA-GL12 (1 mM)
**Fungal strains**		
*C. albicans* (ATCC 14053)	+	-
**Gram-positive bacteria**		
*S. epidermidis* (ATCC 12228)	+	-
*S. haemolyticus* (ATCC 29970)	+	-
*B. subtilis* (ATCC 19659)	+	-
**Gram-negative bacteria**		
*E. coli* (ATCC 25922)	+	-

‘+’: Possessing antimicrobial activity. ‘-’: Possessing no antimicrobial activity. Abbreviation, Amp: ampicillin.

### Stability of OA-GL12

**Figure 11:**
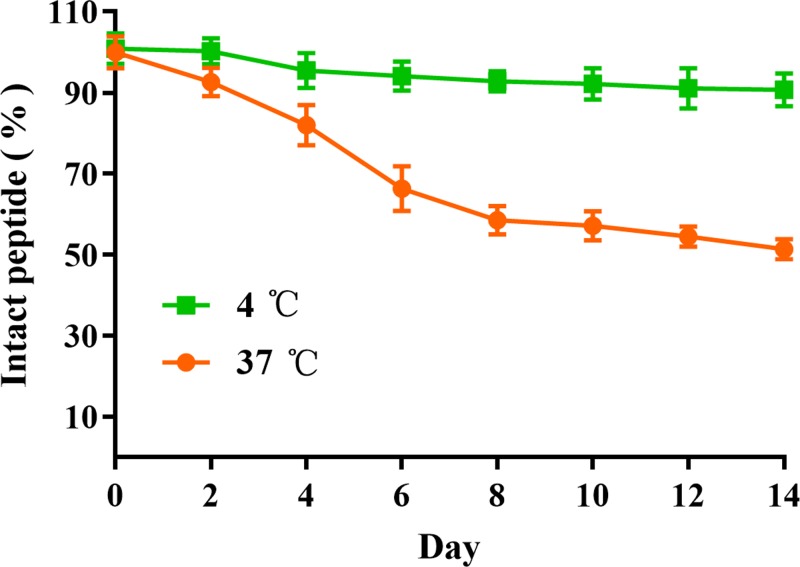
Stability of OA-GL12 Tubes containing OA-GL12 (10 μg/ml dissolved in deionized water) were stored at 4 and 37°C for appointed days. After centrifugation (12000×***g***, 20 min), supernatants were analyzed by C18 RP-HPLC. The initial and residual levels of OA-GL12 were quantitated from peak areas. OA-GL12 was highly stable (4°C) within 14 days and decreased continually until 51.33 ± 1.45% at day 14 (37°C). Data were presented as mean ± S.D., *n*=3.

The stability of OA-GL12 was further performed. As illustrated in Figure 11, OA-GL12 at 4°C did not decrease obviously within 14 days, which suggested that OA-GL12 was constantly stable at storage temperature (4°C). However, OA-GL12 continued its decline at 37°C and then stabilized at approximately 50% from days 8 to 14. Results indicated that OA-GL12 had a high stability at 4°C, rather than 37°C. In addition, the peptide applied to the skin was similar to the stability at 37°C. Results showed that the intact peptide percentage of OA-GL12 was approximately 80–100% in 4 days, and wounds were treated twice daily with samples. So the peptide was considered stable in skin.

## Discussion

Wound healing remains a challenge in clinic [8]. Therefore, accelerating the wound healing is vital to the body. However, wound healing drugs reported, including growth factors, cytokines, and other immunomodulatory factors, do not reach ideal clinic effect [7]. Due to their disadvantages, high cost, low activity and delivery, high-efficiency drugs for wound healing are still urgently needed to be explored. Bioactive peptides, highly active, specific and stable, have provoked worldwide scientific attention [8–10].

Amphibian’s skin secretions possess considerable bioactivities, including antioxidant, antimicrobial, and wound healing promoting abilities [23,35,36]. In this study, a wound healing-promoting peptide (OA-GL12) was identified from skin of *O. andersonii* by employing genomic method (Figure 1). Notably, by BLASTp in NCBI, though its precursor showed high similarity with those frog antimicrobial peptides, such as Nigrocin-2S (Figure 1B), the mature sequence of OA-GL12 was found to be structurally different from other peptides and thus considered to be novel. In addition, differing from Nigrocin-2S composed of an intramolecular disulphide bond, OA-GL12 contained no cyclic motif. Compared with reportedly wound-healing peptides identified from amphibians, such as Cathelicidin-OA1, Cathelicidin-NV, OA-GL21, the mature peptide sequence of OA-GL12 was much shorter and only composed of 12 amino acid residues [13,14,37]. Another short peptide, CW49 with sequence of ‘APFRMGICTTN’, accelerating diabetic wound healing, had a cysteine located at the ninth residue, while the cysteine of OA-GL12 located at the twelfth [38]. Thus, OA-GL12 was a novel wound-healing peptide with a unique structure compared with reportedly peptides from amphibians.

Wound repair requires a highly complicated process, including generally three evolutionarily conserved phases, inflammatory reaction, proliferation, and tissue reconstruction [3,37]. Two types of cells, keratinocytes and fibroblasts play crucial roles in the healing process of wounds [39]. In addition, structural and functional reconstruction and contraction of differentiated myofibroblasts in granulation tissue were necessary in wound healing processes [40,41]. In this study, OA-GL12 obviously accelerated the healing of HaCaT and HSF cell scratches in both time- and concentration-dependent manner *in vitro* (Figures 2–5), but showed little cell proliferative activity. The specific mechanism of cells migration contributing to the wound healing of OA-GL12 still needed to be further explored. According to previous studies, EGFR transactivation, signaling intermediates ERK1/2 and Smad2, the PI3K/AKT and JNK pathways were potential explanation related to HaCaT cell migration [20,25–28]. Additionally, NF-κB and ERK pathways might contribute to the migration capacity of HSF cells [29]. Of note, OA-GL12 strongly accelerated skin wound healing in dose-dependent manner on full-thickness skin-wound mice models (Figure 6). Specially, OA-GL12 (0.1 nM) shared similar effect with KFX (1 mg/ml) *in vivo* (Figure 6B). At postoperative day 10, the rate of re-epithelialization with OA-GL12 (10 nM) was complete while the saline group was still unsatisfactory (Figure 7A,B,D). As illustrated in Figure 7A,C, OA-GL12 accelerated granulation tissue formation in the early stage but contraction in the later period of skin wound healing, which might be due to fibroblast-to-myofibroblast transition regulated by the expression of α smooth muscle actin (α-SMA), the marker of myofibroblast differentiation [42].

Generally, the inflammation reaction is an integral process of wound repair, which has participation of various inflammatory cells and pro-inflammatory cytokines. Amongst them, macrophages produce many cytokines associated with wound healing, such as TNF, IL-1, IL-6, and TGF-β1 [43]. TNF can recruit timely various inflammatory cells, macrophages, which release more TGF-β1 for effective repair. In current study, OA-GL12 induced the secretion of TNF in a concentration-dependent manner in the culture supernatants of RAW264.7 (Figure 8B). TGF-β1, other pro-inflammatory cytokine, participates crucially in inflammation, angiogenesis, re-epithelialization, and connective tissue regeneration. What is more, TGF-β1 helps monocytes convert into macrophages in return, then accelerates the remodeling of granulation tissue and releasing of growth factors contributing to wound healing, which promotes extracellular matrix (ECM) synthesis and wound contraction [44]. ELISA quantitation indicated that OA-GL12 induced the secretion of TGF-β1 on RAW264.7 remarkably even at a low concentration (2 pM), which might explain well tissue reconstruction on mice model in OA-GL12 group (Figures 6–8). These results demonstrated that OA-GL12 might rely on the mechanism of accelerating expression of TNF and TGF-β1 to accelerate wound repair. We assumed that OA-GL12 might recruit much more macrophages to the wound site by the release of TNF and thus induce the release of TGF-β1 remarkably. In addition, more mechanisms underlying of accelerating wound healing was elucidated in this research. According to previous reports, EGFR is one of the most important pathways that stimulate epidermal cell growth and play an important role in skin homeostasis [45–47]. The detailed molecular feature of the EGFR signaling pathway might relate to the activation of MAPK/ERK, PI3K/AKT, and JAK/STAT pathways [48,49]. In this study, results demonstrated that EGFR might make a great contribution to the scratch-healing accelerating activity of OA-GL12 (Figure 9).

Differing from other bioactive peptides having damaging effects on normal cells in previous studies [50,51], OA-GL12 did not show the acute toxicity of human erythrocyte hemolysis and animal level (Tables 1 and 2). Due to its precursor showed high similarity with those frog antimicrobial peptides, such as Nigrocin-2S (Figure 1B), we tested antimicrobial activity and free radical scavenging assay of OA-GL12. OA-GL12 showed no direct antimicrobial activity (Table 3). However, OA-GL12 showed significant free radical scavenging activity (Figure 10), which might also contribute to its promoting wound-healing capacity.

## Conclusion

In the present research, we identified a novel peptide OA-GL12 from the skin of amphibian *O. andersonii*. OA-GL12, with a mature sequence of ‘GLLSGINAEWPC’, could accelerate wound healing at actual concentrations and thus was considered as one of the most powerful wound-healing accelerating agents. The underlying mechanism was also explored and results revealed that OA-GL12 might recruit much more macrophages to the wound site and induce the release of prohealing TGF-β1 remarkably, moreover, EGFR made a great contribution to the scratch-healing accelerating activity of OA-GL12. Additionally, OA-GL12 showed antioxidant activity, but no direct antimicrobial activity, hemolytic or acute toxicity. All these results suggested that OA-GL12 could be a potential template for the development of prohealing agents.

## Abbreviations

ABTS: 2,2′-azino-bis (3-ethylbenzothiazoline-6-sulphonic acid)
DMEM: Dulbecco’s modified Eagle’s medium
DPPH: 2,2-diphenyl-1-picrythydrazyl
EGFR: epidermal growth factor receptor
ERK: extracellular signal-regulated kinase
HaCaT: human keratinocyte
HSF: human skin fibroblast
HUVEC: human umbilical vein endothelial cell
H&E: Hematoxylin and Eosin
i.p.: intraperitoneal injection
IL: interleukin
JAK: Janus kinase
JNK: c-Jun N-terminal kinase
KFX: KangFuXin
MAPK: mitogen-activated protein kinase
MMC: mitomycin C
MTS: 3-(4,5-dimethylthiazol-2-yl)-5-(3-carboxymethoxyphenyl)-2-(4-sulfophenyl)-2H-tetrazolium
NF-κB: nuclear factor κ B
OA-GL12: OA: abbreviation of species (*O. andersonii*), GL: two initial amino acids, 12: peptide length
OD: optical density
PI3K/AKT: phosphatidylinositol 3-kinase/protein kinase B
RAW264.7: murine macrophage cell line
Smad: Mother against decapentaplegic homoing
STAT: signal transducer and ativator of transcription protein
TGF-β1: transforming growth factor β1
TNF: tumor necrosis factor
